# Efficacy of advanced hybrid closed loop systems in cystic fibrosis related diabetes: a pilot study

**DOI:** 10.3389/fendo.2024.1347141

**Published:** 2024-06-20

**Authors:** Marta Bassi, Daniele Franzone, Francesca Dufour, Giordano Spacco, Federico Cresta, Giuseppe d’Annunzio, Giacomo Tantari, Maria Grazia Calevo, Carlo Castellani, Nicola Minuto, Rosaria Casciaro

**Affiliations:** ^1^ Pediatric Clinic, Endocrinology Unit, Istituto di Ricovero e Cura a Carattere Scientifico (IRCCS) Istituto Giannina Gaslini, Genoa, Italy; ^2^ DINOGMI - Department of Neuroscience, Rehabilitation, Ophthalmology, Genetics, Maternal and Child Health, University of Genoa, Genoa, Italy; ^3^ Epidemiology and Biostatistics Unit, Scientific Directorate, IRCCS Istituto Giannina Gaslini, Genoa, Italy

**Keywords:** AHCL (Advanced Hybrid Closed Loop), cystic fibrosis, CFRD (cystic fibrosis related diabetes), CGM (continuous glucose monitoring), insulin pumps, time in range (TIR)

## Abstract

**Background and aims:**

Cystic fibrosis related diabetes (CFRD) is correlated with worsening of nutritional status and greater deterioration of lung function. The role of new technologies for the treatment of CFRD is little explored. The aim of the study was to evaluate the efficacy of Advanced Hybrid Closed Loop (AHCL) systems on glycemic control in CF patients.

**Methods:**

A single-center retrospective study on CFRD patients using AHCL systems was performed. Glycated hemoglobin (HbA1c) values and Continuous Glucose Monitoring (CGM) metrics were collected at T0 (AHCL placement), T1 (1-month), T2 (6-months) and T3 (1-year) to evaluate glycemic control.

**Results:**

10 patients were included in the study. Data showed a reduction of HbA1c value (7.31 ± 0.34 to 6.35 ± 1.00; p=0.03), glycemic variability (p=0.05) and insulin requirement (p=0.03). The study population reached American Diabetes Association (ADA) recommended glycemic targets at 1-year. An increase in the Time in Range (TIR) and a reduction in time in hyperglycemia were also observed, although not statistically significant.

**Conclusions:**

In patients with CFRD, the use of AHCL leads to an improvement in glycemic control in terms of HbA1c and glycemic variability. The increase in TIR and the reduction of time in hyperglycemia, although not statistically significant, are extremely encouraging from a clinical point of view. Further studies with a larger population and a longer follow-up are needed. The results of this study demonstrate the importance of proposing the use of AHCL even in CF patients, who could benefit from glycemic improvement also in terms of nutritional status and respiratory function.

## 1 Introduction

Cystic fibrosis-related diabetes (CFRD) is one of the most common extrapulmonary manifestations of cystic fibrosis (CF) which affects up to 20–30% of adolescents and 30–50% of young adults living with CF (1, 2). The diagnosis of CFRD can be made in CF patients according to the American Diabetes Association (ADA) criteria. ADA Clinical Practice Guideline recommends patients with cystic fibrosis to perform CFRD annual screening with oral glucose tolerance test (OGTT), starting from the age of 10 (3). A poor glycemic control has been related to a more severe clinical outcome, characterized by the progression of lung function deterioration and poorer nutritional status, resulting in a higher risk of recurrent pulmonary exacerbations, chronic growth of respiratory pathogens and earlier mortality (4–6).

Cornerstones of CFRD management are glucose monitoring and insulin therapy, which is the only treatment currently approved for CFRD (7). Self-monitoring of blood glucose (SMBG) multiple times a day can be burdensome and difficult for many patients (8).

Huge technological advancements in diabetes management have been achieved during the past decade, such as the development of the modern flash/continuous glucose monitoring (FGM/CGM), insulin pumps, and automated insulin delivery (AID) systems, creating a paradigm shift in Type 1 Diabetes Mellitus (T1DM) standards of care (9), although the impact of these devices in individuals with CFRD is less clear (10). FGM and CGM systems are minimally invasive devices tracking glucose levels continuously. Glucose readings are sent to a smart device in real-time for CGM or on-demand for FGM. CGM allowed the development of the Sensor Augmented Pump (SAP), consenting the association of the two systems without providing any interaction between glucose sensor and insulin pump. Subsequently, SAPs were developed with the Low Glucose Suspend (LGS) and Predictive Low Glucose Suspend (PLGS) function, automatically interrupting the basal insulin infusion in case of hypoglycemia or predicted hypoglycemia. In 2015 Hybrid Closed Loop (HCL) systems were introduced as integrated algorithms which automatically regulate basal insulin delivery based on CGM glucose values. In 2019 the Advanced Hybrid Closed Loop (AHCL) were developed combining automated basal rate and correction boluses to keep glycemic values in a target range (11).

The application of diabetes technology in CF patients has consistently increased during the last years. In 2009, CGM systems were validated for this population of patients (12). Subsequent studies demonstrated that CGM measurements of hyperglycemia and glycemic variability were superior to HbA1c in distinguishing patients with and without CFRD (13). Adjustment of insulin treatment based on CGM metrics was associated with improvements in lung function, weight and reduced pulmonary function decline (14). Regarding use of insulin pumps in CFRD, there is lack of evidence. The studies performed, excluding case reports (15, 16), demonstrated CSII and SAP safety and efficacy for treatment of CFRD (17, 18). There are no studies exploring the benefit of LGS or PLGS systems in CFRD (10).

In the last two years, the use of AHCL systems, initially developed for T1DM treatment, has been extended to other forms of diabetes and special populations, such as patients affected by CFRD (11). A small pilot study on three patients showed treatment satisfaction, reduced burden of diabetes care and a reduction in glycemic variability (19). The first study to report a beneficial effect of AHCL technology (Tandem Control-IQ algorithm) on glycemic control in adults and adolescents with CFRD was performed by Scully et al. in 2022. An improvement in glycemic control as well in glycemic variability were observed (20).

## 2 Methods

### 2.1 Aims of the study

The aim of this study was to evaluate the efficacy of AHCL systems in CF patients in terms of HbA1c and CGM metrics over a 1-year follow-up period.

The primary aim was to evaluate the improvement of glycemic control in terms of glycated hemoglobin (HbA1c) in CFRD patients using AHCL. Secondary aims were the evaluation of the improvements in CGM metrics, the evaluation of changes in weight, BMI, insulin requirement and FEV1%, the achievement of ADA recommended targets and the safety of the system in terms of occurrence of severe hypoglycemia (SH) episodes.

### 2.2 Population characteristics

A retrospective single center study involving a cohort of patients affected by CFRD followed by the Regional Cystic Fibrosis Center and Regional Pediatric Diabetes Center of IRCCS Giannina Gaslini (Genoa) was performed. All patients affected by CFRD using AHCL systems for at least 1-year, independently from previous therapy, were included. Data collection and subsequent analysis were conducted in 2022–2023.

Because of the retrospective nature of the study the ethic approval and informed consent already signed by patients at the disease onset and renewed yearly, in which they agree on the use of clinical data for research purposes, were used. In addition, all patients provided a specific informed consent for the collection of data.

### 2.3 AHCL systems

Two different AHCL systems were used by the study population: the Tandem t:slim X2 Control IQ™ system (Tandem Inc., San Diego, California) and the Minimed™ 780G system (Minimed Medtronic, Northridge, California). The two systems differ in the type of algorithm and in some features, but both are able to automatically adjust basal insulin delivery in relation to the glucose level detected by the CGM, suspend insulin delivery in the event of hypoglycemia (current or predicted) and deliver automatic corrective boluses in case of hyperglycemia. The use of AHCL systems in patients affected by CFRD is part of our clinical practice and the choice of the device depends on the specific needs of the single patient. For this reason and given the retrospective nature of the study, a single AHCL system was not used for the study.

### 2.4 Clinical and CGM data collection

Data were collected at T0 (starting of AHCL system), T1 (1-month after starting AHCL system), T2 (6-months after starting AHCL system) and T3 (1-year after starting AHCL system). Clinical data were collected from electronic clinical records of regular follow-up visits and included age, gender, age at CFRD diagnosis, age at insulin therapy initiation, duration of CFRD, bacterial colonization, FEV1% predicted, weight, BMI, eventual therapy with CFRD modulator drugs or glucocorticoid, lung transplant status, pancreatic insufficiency, previous diabetes treatment, insulin requirement and glycated hemoglobin (HbA1c). Where possible, FGM or CGM data were obtained in a 14-day period within one month from T0.

FGM/CGM metrics included: Time in Range (TIR, 70–180 mg/dl), Time above Range (TAR, 180–250 mg/dl), TAR>250 (>250 mg/dl), Time below Range (TBR, 54–70 mg/dl) and TBR<54 (<54 mg/dl), average glucose (AG) value, standard deviation (SD), glucose coefficient of variation (CV) and percentage of sensor use (%). FGM/CGM and insulin pump data were collected remotely, with real time glucose data sharing dedicated platforms or by downloading them and storing them on cloud platforms available at our center.

Data collected at T1, T2 and T3 were HbA1c and CGM metrics. Additionally, at T2 and T3 weight, BMI, insulin requirement (total daily insulin dose - U/kg/day) and FEV1% predicted were collected. Hospitalization in the 1-year period before T0 and T3 were recorded.

### 2.5 Statistics

Data are described as mean and standard deviation (SD) or median and range for continuous variables, and as absolute and relative frequencies for categorical variables. The Kolmogorov-Smirnov test was used to establish the normality of continuous variables. Comparisons between T0, T1, T2 and T3 to examine continuous variables were performed using Paired Wilcoxon test. P values ≤ 0.05 were considered statistically significant, and all P values were based on two tailed tests. Statistical analysis was performed using SPSS for Windows (SPSS Inc, Chicago, Illinois USA).

## 3 Results

Population characteristics at baseline are summarized in **Table 1**. Ten patients with CFRD, on insulin therapy with AHCL systems (5 on Tandem Control IQ™ and 5 on Minimed™ 780G) were included in the study, 3 (30%) of them were female and 7 (70%) had at least one copy of F508del mutation. Mean age was 39.3 years (range 18.4–50.1 years), mean FEV1 was 80% ± 29.6% and 9 patients (90%) had a mild or moderate lung disease (FEV1 > 80% of predicted as mild disease and FEV1 between 50% and 80% for moderate lung disease). Three patients had previously undergone a lung transplant and were on corticosteroid therapy; none of the other patients were on steroid therapy during the study period. Mean HbA1c value was 7.31% ± 0,34%, only 2 patients (20%) met recommended value of <7.0%.

**Table 1:** Population characteristics at baseline (T0).

	*Total (n = 10)*
Age (years)	39.3 ± 12.7
Female	3 (30%)
B.M.I. (Kg/m^2^)	22.9 ± 3.1
Age at CFRD diagnosis (years)	21.3 ± 7.9
Duration of CFRD (years)	17.8 ± 10.6
HbA1c (%)	7.31 ± 0.34
Genotype	
F508del homozygous	5 (50%)
F508del heterozygous	2 (20%)
Other	3 (30%)
Bacterial colonization	
OXA-S *S. aureus*	5 (50%)
*P. aeruginosa*	2 (20%)
OXA-S *S.aureus and P. aeruginosa*	2 (20%)
OXA-S *S.aureus and B. cepacia*	1 (10%)
Pancreatic insufficiency	10 (100%)
CF-related liver disease	0 (0%)
Lung Transplant (on CCS therapy)	3 (30%)
FEV 1 (% predicted)	79.90 ± 29.62
Hospitalizations due to CF exacerbations in the previous 12 months	6 (60%)
CFTR modulator therapy	
None	5 (50%)
Ivacaftor–Lumacaftor	2 (20%)
Elexacaftor–Tezacaftor–Ivacaftor	3 (40%)
Diabetes treatment	
MDI	4 (40%)
Conventional insulin pump	3 (30%)
PLGS	3 (30%)
Glycemic monitoring	
SMBG	1 (10%)
FGM	5 (50%)
CGM	4 (40%)

BMI, Body Mass Index.

CFRD, Cystic Fibrosis Related Diabetes.

HbA1c, Glycated hemoglobin.

OXA-S, Oxacylline sensible.

MFDI, Multiple Daily Injections.

PLGS, Predictive Low Glucose Suspend.

SMBG, Self Monitoring Blood Glucose.

FGM, Flash Glucose Monitoring.

CGM, Continuous Glucose Monitoring.

CCS - Corticosteroids.

**Table 2** reports HbA1c, weight, BMI, insulin requirement and CGM metrics expressed as mean values and standard deviations (SD), at baseline and at 1-month, 6-months, and 1-year from transition to AHCL system. CGM metrics at baseline were available for 8 patients, one patient did not have available 1-month follow-up data and one patient did not have available 1-year follow-up data. HbA1c value of one patient was only recorded at twelve months.

**Table 2:** CGM metrics, HbA1c, weight, BMI, FEV1 and insulin requirement at T0, T1 (1 month), T2 (6 months) and T3 (1 year) after initiation of AHCL system.

	T0	T1	*p (T1vsT0)*	T2	*p (T2vsT0)*	T3	*p (T3vsT0)*
**HbA1c %**	7.31 ± 0.34	6.81 ± 0.42	0.07	6.57 ± 0.85	**0.01**	6.35 ± 1.00	**0.03**
**TIR% (70–180 mg/dL)**	60.0 ± 20.0	68.71 ± 16.91	0.17	76.29 ± 13.30	0.06	76.17 ± 13.66	0.34
**TAR% (181–250 mg/dL)**	22.71 ± 9.39	22.43 ± 10.01	0.53	18.71 ± 10.14	1	18.67 ± 11.55	0.60
**TAR% (>250 mg/dL)**	15.00 ± 9.93	8.43 ± 8.14	0.14	4.29 ± 3.64	0.06	3.83 ± 4.07	0.07
**TBR% (55–69 mg/dL)**	1.71 ± 2.14	0.29 ± 0.49	0.07	0.57 ± 0.53	0.13	0.83 ± 0.75	0.27
**TBR% (<54 mg/dL)**	0.43 ± 0.79	0	0.18	0.03 ± 0.07	0.28	0.33 ± 0.52	0.70
**AG (mg/dL)**	169.71 ± 32.4	158.86 ± 27.46	0.61	147.57 ± 21.4	0.13	149.0 ± 25.27	0.50
**SD (mg/dl)**	66.0 ± 17.78	53.50 ± 12.45	0.07	53.67 ± 7.57	0.28	47.00 ± 11.31	0.18
**CV (%)**	39.00 ± 5.63	33.31 ± 2.94	**0.02**	31.44 ± 3.44	**0.04**	30.23 ± 4.16	**0.05**
**Weight (kg)**	63.71 ± 11.63			65.69 ± 11.79	0.06	64.30 ± 1.16	0.11
**BMI**	22.95 ± 3.08			23.66 ± 3.08	0.08	23.38 ± 2.91	0.17
**TDI dose (U/kg/day)**	0.59 ± 0.29			0.51 ± 0.21	**0.03**	0.50 ± 0.21	**0.03**
**FEV1% predicted**	79.90 ± 29.62			79.40 ± 30.51	0.81	82.56 ± 30.23	0.81

HbA1c, Glycated Hemoglobin.

TIR, Time in Range.

TAR, Time Above Range.

TBR, Time Below Range.

AG, Average Glucose.

SD, Standard Deviation.

CV, Coefficient of Variation.

BMI, Body Mass Index.

TDI, Total Daily Insulin.

Bold, statistically significant.

**Figure 1:**
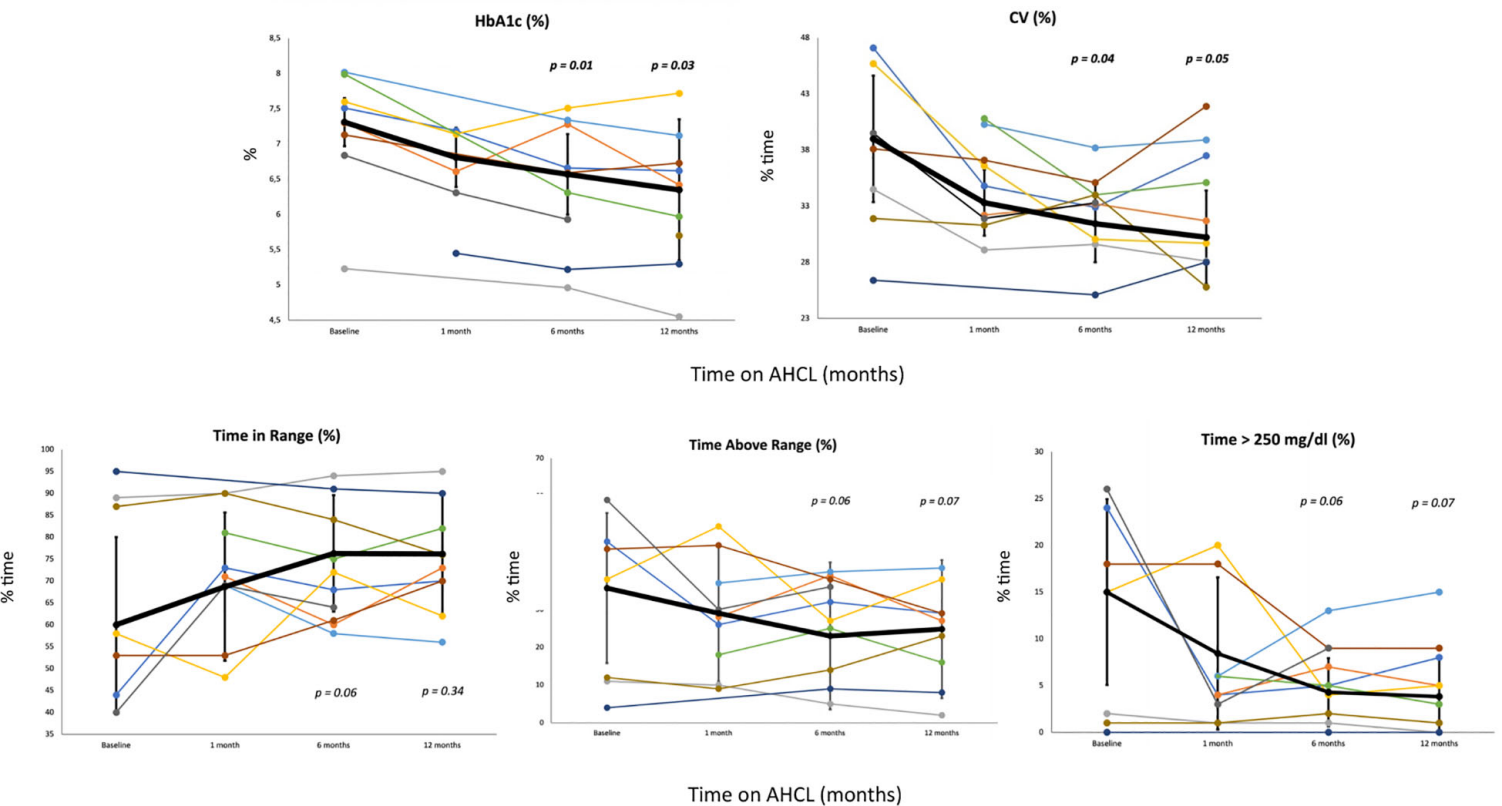
Change in key CGM measures from baseline to 1, 6 months and 12 months after starting AHCL. The figure depicts the change in five key glycemic variables (HbA1c, CV, % time in range, % time above range and % time > 250 mg/dl) with each patient represented by a different color. Thicker black lines represent the least LS mean across all subjects with the error bars depicting the SE. (HbA1c, glycated hemoglobin; CV, coefficient of variation CGM, continuous glucose monitor; LS, least squares; SE, standard error).

HbA1c showed a statistically significant reduction over the 1-year study period (7.31 ± 0.34 to 6.57 ± 0.85 at T2; p=0.01, to 6.35 ± 1.00 at T3; p=0.03). CV showed a statistically significant reduction at 1-month, 6-months, and 1-year from starting of AHCL (39.00 ± 5.63 to 31.44 ± 3.44 at T2; p=0.04, to 30.23 ± 4.16; p=0.05). Total daily insulin requirement (U/kg/day) decreased significantly during the study period (0.59 ± 0.29 to 0.51 ± 0.21 at T2; p=0.03, to 0.50 ± 0.21 at T3; p=0.03). A trend in increase in TIR during the one-year study period was observed (60.0 ± 20.0 to 76.29 ± 13.30 at T2; p=0.06, to 76.17 ± 13.66 at T3; p=0.34). In addition, we reported a trend in reduction in % time in hyperglycemia > 250 mg/dl (15.0 ± 9.93 to 4.29% ± 3.64 at T2; p=0.06, to 3.83 ± 4.07 at T3; p=0.07). No significant difference of time in hypoglycemia was observed from baseline to 1-year. After 6-month and 1-year from transition to AHCL system, the study population (expressed as mean values) achieved ADA-recommended CGM-based glycemic targets (21), only minimally reached at T0 (**Table 3**). **Supplementary Table 1** shows the increase in the number of patients reaching the over mentioned targets. Variation in HbA1c and CGM metrics across the six-month study period are presented for each patient in **Figure 1**. No significant differences were found between T0 and T3 in terms of FEV1%, and BMI. However, BMI increased from 22.95 ± 3.08 to 23.38 ± 2.91 (p=0.17). The number of hospitalizations per patient for CF exacerbations decreased from 0.56 ± 0.73 in the year before T0 to 0.11 ± 0.33 during the 1-year follow-up period (p=0.05). No severe hypoglycemia (SH) events occurred between T0 and T3. At the time of data analysis, all the participants were still on AHCL therapy, with a median of duration of use of 26.23 months (range 17.39 – 37.65 months). The results stratified by type of AHCL used (Minimed 780G and Tandem Control-IQ) are shown in **Supplementary Table 2**.

**Table 3:** Achieving ADA-Recommended Continuous Glucose Monitor Targets at Baseline and after 6 months and 1 year from starting AHCL system (20) presented as medium population values.

	Recommended	T0		T2		T3	
**HbA1c**	< 7%	7.31%	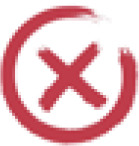	6.57%	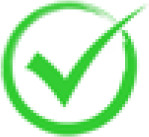	6.35%	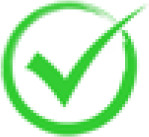
**TIR% (70–180 mg/dL)**	> 70%	60%	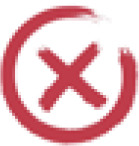	76.29%	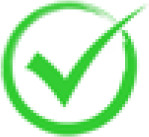	76.17%	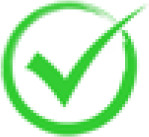
**TAR% (>180 mg/dL)**	< 25%	35.67%	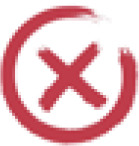	23%	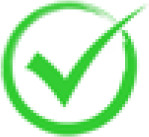	18.67%	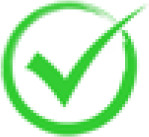
**TAR% (>250 mg/dL)**	< 5%	15%	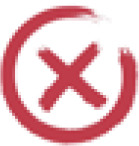	4.29%	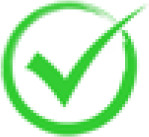	3.83%	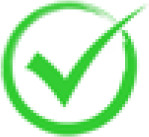
**TBR% (<70 mg/dL)**	< 4%	2.14%	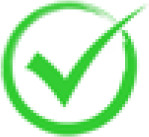	0.6%	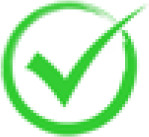	0.83%	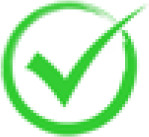
**TBR% (<54 mg/dL)**	< 1%	0.4%	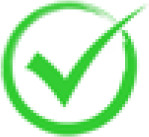	0.03%	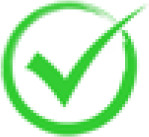	0.33%	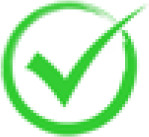
**CV**	< 36%	39%	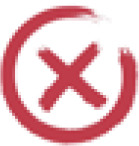	31.44%	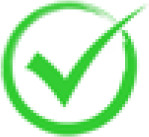	30.23%	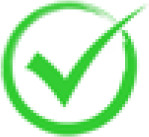

HbA1c, Glycated Hemoglobin.

TIR, Time in Range.

TAR, Time Above Range.

TBR, Time Below Range.

AG, Average Glucose.

SD, Standard Deviation.

CV, Coefficient of Variation.

## 4 Discussion

This study suggests that AHCL systems are effective in improving glycemic control in CFRD patients, reducing HbA1c, CV values and insulin requirement and increasing the proportion of patients reaching ADA recommended CGM-based targets. The efficacy of insulin pumps and AHCL in T1DM are widely described and a consistent number of real-world data studies are available (11). Conversely, few studies exploring the efficacy and safety of insulin pumps in the management of CFRD are available. In 2009, Hardin et al. performed the first study to evaluate the efficacy of continuous subcutaneous insulin infusion (CSII) in a cohort of 9 CFRD adult patients. Results showed a significant improvement in fasting and post-prandial blood glucose levels, HbA1c, body weight and lean mass after 6-months of CSII use (17). In 2023, Grancini et al. demonstrated the improvement of glycemic control parameters and increase in fat mass in 20 patients after 24-months of SAP use (18).

The first application of AHCL technology in CFRD was a three-arm random-order crossover pilot study. A closed loop artificial pancreas system, both in bihormonal (insulin+glucagon) and insulin-only configuration was compared with usual diabetes care in 3 adult patients. A non-significant reduction in glycemic variability with mean glucose levels <150 mg/dl and minimal hypoglycemia were observed. Patients reported improvements in treatment satisfaction and decreased treatment burden (19). In 2022 a multicenter retrospective study compared glycemic control at baseline and after one and three months from transition to the AHCL system Tandem t:slim X2 pump with Control IQ^®^ technology in 13 patients with CFRD. A significant increase of 15.2% in Time in Range (TIR) was observed (54.3% to 69.5%, p = 0.001) as well as a decrease in hyperglycemia (TAR – time above range) and glycemic variability (CV – Coefficient of Variation). No significant differences in time spent in hypoglycemia were reported (20).

Given the limited data in literature on the efficacy and safety of AHCL in CFRD and differently from previous study, we performed a single center retrospective study among all CFRD patients referred to our Cystic fibrosis and Pediatric Diabetology center using AHCL systems, regardless of the type of system.

We chose the improvement of the HbA1c as primary outcome due to the availability of this data even for those who did not use CGM at T0. Data showed that the transition to an AHCL system is associated with a significant reduction in HbA1c and glycemic variability (CV). Clinically relevant trends in TIR improvement (+16%) and in reduction in TAR>250 mg/dl (-11%) were also observed, although probably due to the small sample size, results were next to statistical significance for both. Furthermore, the significant progressive reduction observed in insulin requirement (-0.09 U/kg/day, p = 0.03) demonstrates that the improvement in glycemic control is not due to an increase of TDI but rather to the optimization of insulin therapy. The use of insulin pumps leads to a reduction in daily insulin requirements also in T1DM (21). Nevertheless, after starting an AHCL systems the optimization of insulin therapy seems to be related to stability or increase in insulin requirement, in particular due to an increase in the percentage of bolus insulin and a reduction in the percentage of basal insulin dose (22, 23). The percentage of time in hypoglycemia did not increase with the introduction of the AHCL system in our cohort of patients, confirming not only the efficacy, but also the safety in the use of these devices in CFRD patients. Considering the average of the CGM metrics reached by the study population, the great efficacy of AHCL on glycemic control is demonstrated by the achievement of all CGM-based recommended targets at T2 and T3: TIR >70%, TAR<25% and TAR>250 mg/dL <5%, TBR< 4% and TBR<54 mg/dL <1% (24). Most recommended targets were not achieved with the other types of insulin therapy previously used (**Table 3**).

Considering how AHCL algorithms work, it is important to underline the pathophysiological differences of CFRD and T1DM in terms of insulin deficiency. In case of meal insulin bolus omission, the algorithm increases the insulin infusion rate driven by CGM sensor glucose value; it could also deliver a correction bolus in case the increment in basal insulin rate is not sufficient. This is effective for individuals with T1DM with complete insulin deficiency. CFRD is firstly characterized by impaired insulin secretion and progressive islet cell damage with insulin insufficiency developing over time. In addition, insulin resistance related to chronic inflammation, cyclic infections, glucocorticoid therapy and an association with genetic predictors of Type 2 Diabetes Mellitus (T2DM) is associated (25). In patients with CFRD, the residual endogenous insulin production alongside increased insulin delivered by the insulin pump can lead to reactive post-prandial hypoglycemia. Reactive hypoglycemia is a common side effect observed in CFRD, as a result of delayed first phase insulin secretion and late compensatory second phase insulin secretion (26). Pancreatic insufficiency, despite a correct enzyme replacement therapy, can lead to fat malabsorption, more rapid gastric emptying, and more significant post-prandial hyperglycemia (27). Further complicating CFRD management, gastroparesis has been estimated to occur in approximately one third of CF patients (28). Hence, in patients with CFRD it may be even more important to respect the correct timing of the bolus, which must always be performed before meals. In this regard, it would also be interesting to study the glycemic trend of CFRD patients using AHCL who omit meal boluses, as done for patients with T1DM (29). Some authors agree on starting an AID therapy with less aggressive correction if automated correction boluses are provided by the system (10). Lower basal rates in the overnight hours may also be required for CFRD patients with significant endogenous insulin secretion (30).

Time spent in hypoglycemia did not increase using AHCL and no cases of severe hypoglycemia (SH) occurred; these findings demonstrate the safety of these devices even in this form of diabetes which is different from T1DM.

Five patients were already on modulator therapy when they started AHCL systems, of whom three on elexacaftor-tezacaftor-ivacaftor (ETI). It is still controversial if and how much these therapies impact on CFRD. Preliminary data have shown improvements in average glucose levels and reduced CV following ETI treatment, but no significant changes in insulin total daily dose (31). An observational study of 134 adult patients treated with ETI found a random improvement of glucose and HbA1c levels in patients without CFRD but not in those with CFRD (32). Recently, Grancini et al. demonstrated a decrease of HbA1c and glycemic variability and an increase of fat mass after six months of ETI treatment (33). Due to the small number under treatment, this study could not contribute with regard to the effects of ETI on glycemic improvements.

Even though CFRD is the most common comorbidity in CF, many patients are unaware of the possibility to develop it and CFRD diagnosis may be seen as a further increase in therapy burden, which is already a complex, time-consuming medical regimen involving airway clearance, inhaled therapies and antibiotics, enzyme replacement and caloric supplementation (34, 35). The use of AHCL systems in T1DM has been associated with an improvement in Quality of Life (QoL), quality of sleep and reduced impact of diabetes on daily life (36, 37). Despite perceived benefits, the use of diabetes technology in people with Cystic Fibrosis is still low and related patients’ perception is still understudied. In a 2021 survey of CFRD patients in the United States, 75% of youth and adults reported CGM use, similar to T1DM patients, while only 29% reported insulin pump use (38). A significant benefit from CGM use was reported, but also a greater burden from insulin pump use. In addition, high device discontinuation rates were observed: 19% for CGM and 28% for insulin pump, most commonly due to increased concerns about glycemia, cost and pain related to the device use. Considering our study population all the participants were still on insulin therapy with AHCL and many of them over two years after the start of the system; the long-term adherence reported should be encouraging for CF centers to propose automated insulin delivery systems for their insulin-dependent patients. A prospective study evaluating AHCL treatment satisfaction in CF patients would be beneficial.

Advanced therapeutic solutions should be proposed to insulin-dependent CF patients by diabetologists experts in technological field, along with a close follow-up by a specialized multidisciplinary team with expertise in diabetes and CF; this approach can lead to a larger use of these advanced tools, an improvement of glycemic control and a low discontinuation rate in CF patients (39). As stated by “JDRF Barriers and Drivers to technology”, the first reason for patients not using technological devices is that the clinician did not recommend it (40). Further studies with a greater number of patients and a longer follow-up period are needed; our results show the importance to offer AHCL systems to this population of patients which could benefit from glycemic improvement as well as in nutritional status and respiratory function.

The evaluation of treatment efficacy in terms of CGM metrics, the application of different AHCL systems and the single center data collection are the strengths of this study, although several limitations must be assessed. The relatively small number of patients and a low power of the study related with the low rate of use of technology in CFRD, although still adequate to detect significant changes in some glycemic measures, should be considered as a limitation. Furthermore, the retrospective nature of the study led to the difficulty to obtain complete clinical and CGM data at baseline in patients who were not on CGM prior to starting the AHCL system. A possible consequence of this limitation is the difference of statistical significance between the improvement observed in HbA1c values and CGM metrics.

## 5 Conclusions and future perspectives

In conclusion, AHCL systems showed to be effective in improving glycemic control in CFRD patients, reducing HbA1c, CV values and insulin requirement and increasing the proportion of patients reaching ADA recommended CGM-based targets. The long-term adherence to AHCL treatment observed in CF patients is encouraging for CF centers to propose these systems for their insulin-dependent patients. Multidisciplinary teams should support the use of technological devices for CFRD treatment, associated with a successful and close collaboration of each specialist during follow-up. Prospective study evaluating AHCL treatment satisfaction in patients affected by CFRD and evaluating the efficacy and safety of these systems on a higher number of patients and a longer follow-up would be very useful.

## Data availability statement

The original contributions presented in the study are included in the article/**Supplementary Material**. Further inquiries can be directed to the corresponding author.

## Author contributions

MB: Writing – original draft, Conceptualization. DF: Writing – original draft, Data curation. FD: Writing – review & editing, Data curation. GS: Writing – review & editing, Data curation. FC: Writing – review & editing. GD: Writing – review & editing. GT: Writing – review & editing. MC: Writing – original draft, Formal Analysis. CC: Writing – review & editing, Supervision. NM: Writing – original draft, Supervision, Conceptualization. RC: Writing – review & editing, Supervision.
