# Supplementary material for: Efficacy of advanced hybrid closed loop systems in cystic fibrosis related diabetes: a pilot study
Source: Front Endocrinol (Lausanne). 2024 Jun 20;15:1347141. doi: 10.3389/fendo.2024.1347141 (PMC11222316; doi:10.3389/fendo.2024.1347141)
Supplement: Supplementary file 1 [file Table_1.docx]

**Supplementary Table 1.** Number of patients achieving ADA-Recommended Continuous Glucose Monitor Targets at Baseline, after 6 months and after 1 year from starting AHCL system (21)

|  | **T0, Baseline**  **N = 8** | **T2, 6 Months**  **N = 10** | **T3, 12 Months**  **N = 9** |
| --- | --- | --- | --- |
| **CV < 36%** | 3 (37.5%) | 5 (50%) | 7 (78%) |
| **% time 70–180 mg/dL > 70%** | 3 (37.5%) | 5 (50%) | 5 (56%) |
| **% time 181-250 mg/dL < 25%** | 5 (62.5%) | 5 (50%) | 6 (67%) |
| **% time >250 mg/dL < 5%** | 3 (37.5%) | 5 (50%) | 4 (44%) |
| **% time 55-69 mg/dL < 4%** | 7 (87.5%) | 10 (100%) | 9 (100%) |
| **% time <54 mg/dL < 1%** | 6 (75%) | 9 (90%) | 5 (56%) |
