# Supplementary material for: Efficacy of advanced hybrid closed loop systems in cystic fibrosis related diabetes: a pilot study
Source: Front Endocrinol (Lausanne). 2024 Jun 20;15:1347141. doi: 10.3389/fendo.2024.1347141 (PMC11222316; doi:10.3389/fendo.2024.1347141)
Supplement: Supplementary file 2 [file Table_2.docx]

**Supplementary Table 2A.** CGM outcomes and HbA1c at T0, T1 (1 month), T2 (6 months) and T3 (1 year) after initiation of AHCL system for Minimed 780G Users.

|  | **T0** | **T1** | **T2** | **T3** |
| --- | --- | --- | --- | --- |
| **HbA1c %** | 7.13 ± 1.09 | 6.98 ± 0.32 | 6.75 ± 1.05 | 6.49 ± 1.19 |
| **TIR% (70–180 mg/dL)** | 63.67 ± 23.03 | 70.33 ±21.13 | 78.00 ±14.00 | 75.67 ±17.21 |
| **TAR% (181-250 mg/dL)** | 18.67 ± 8.39 | 21.00 ± 11.53 | 18.00 ± 12.29 | 18.67 ± 15.63 |
| **TAR% (>250 mg/dL)** | 13.67 ± 11.06 | 8.33 ± 10.21 | 3.33 ± 2.08 | 4.33 ± 4.04 |
| **TBR% (55-69 mg/dL)** | 3.00 ± 3.00 | 0.33 ± 0.58 | 0.67 ± 0.58 | 1.00 ± 1.00 |
| **TBR% (<54 mg/dL)** | 1.00 ± 1.00 | 0 | 0.00 ± 0.00 | 1.00 ± 1.00 |
| **AG (mg/dL)** | 158.67 ± 31.34 | 156.00 ± 34.83 | 144.00 ± 24.25 | 149.0 ± 32.92 |
| **SD (mg/dl)** | - | - | - | - |
| **CV (%)** | 42.43 ± 6.91 | 33.50 ± 3.91 | 30.85 ± 1.79 | 31.77 ± 5.03 |

HbA1c – Glycated Hemoglobin

TIR – Time in Range

TAR – Time Above Range

TBR – Time Below Range

AG – Average Glucose

SD – Standard Deviation

CV – Coefficient of Variation

**Supplementary Table 2B.** CGM outcomes and HbA1c at T0, T1 (1 month), T2 (6 months) and T3 (1 year) after initiation of AHCL system for Tandem Control-IQ Users.

|  | **T0** | **T1** | **T2** | **T3** |
| --- | --- | --- | --- | --- |
| **HbA1c %** | 7.32 ± 0.60 | 6.31 | 6.28 ± 0.33 | 6.00 ± 0.05 |
| **TIR% (70–180 mg/dL)** | 74.00 ± 29.71 | 67.50 ±16.42 | 75.00 ±14.76 | 76.67 ±13.01 |
| **TAR% (181-250 mg/dL)** | 16.00 ± 15.19 | 23.50 ± 10.41 | 19.25 ± 10.21 | 18.67 ± 9.45 |
| **TAR% (>250 mg/dL)** | 9.00 ± 14.73 | 8.50 ± 7.94 | 5.00 ± 4.69 | 3.33 ± 4.93 |
| **TBR% (55-69 mg/dL)** | 1.00 ± 0.00 | 0.25 ± 0.50 | 0.50 ± 0.58 | 0.67 ± 0.58 |
| **TBR% (<54 mg/dL)** | 0.00 ± 0.00 | 0 | 0.05 ± 0.10 | 0.33 ± 0.58 |
| **AG (mg/dL)** | 150.67 ± 51.86 | 161.00 ± 26.18 | 150.25 ± 22.40 | 149.0 ± 22.65 |
| **SD (mg/dl)** | 62 ±29.70 | 53 50±12.45 | 53.67 ±7.57 | 47 ±11.31 |
| **CV (%)** | 32.60 ± 6.58 | 33.17 ± 2.65 | 31.87 ± 4.58 | 28.70 ± 3.31 |

HbA1c – Glycated Hemoglobin

TIR – Time in Range

TAR – Time Above Range

TBR – Time Below Range

AG – Average Glucose

SD – Standard Deviation

CV – Coefficient of Variation
